# Exploring the Predictive Value of Grading in Regions Beyond Peritumoral Edema in Gliomas based on Radiomics

**DOI:** 10.2174/0115734056387494250823132119

**Published:** 2025-08-28

**Authors:** Jie Pan, Jun Lu, Shaohua Peng, Minhai Wang

**Affiliations:** 1 Department of Medical Imaging Center, The First Affiliated Hospital of Medical College, Shihezi University, Medical Imaging Center, Shihezi, China

**Keywords:** MRI, Radiomics, Glioma grading, Peritumoral regions, CNS

## Abstract

**Introduction:**

Accurate preoperative grading of adult-type diffuse gliomas is crucial for personalized treatment. Emerging evidence suggests tumor cell infiltration extends beyond peritumoral edema, but the predictive value of radiomics features in these regions remains underexplored.

**Methods:**

A retrospective analysis was conducted on 180 patients from the UCSF-PDGM dataset, split into training (70%) and validation (30%) cohorts. Intratumoral volumes (VOI_I, including tumor body and edema) and peritumoral volumes (VOI_P) at 7 expansion distances (1–5, 10, 15 mm) were analyzed. Feature selection involved Levene's test, t-test, mRMR, and LASSO regression. Radiomics models (VOI_I, VOI_P, and combined intratumoral-peritumoral models) were evaluated using AUC, accuracy, sensitivity, specificity, and F1 score, with Delong tests for comparisons.

**Results:**

The combined radiomics models established for the intratumoral and peritumoral 1-5mm ranges (VOI_1-5mm) showed better predictive performance than the VOI_I model (AUC=0.815/0.672), among which the VOI_1 model performed the best: in the training cohort, the AUC was 0.903 (accuracy=0.880, sensitivity=0.905, specificity=0.855, F1=0.884); in the validation cohort, the AUC was 0.904 (accuracy=0.852, sensitivity=0.778, specificity=0.926, F1=0.840). This model significantly outperformed the VOI_I model (p<0.05) and the 10/15mm combined models (p<0.05).

**Discussion:**

The peritumoral regions within 5 mm beyond the edematous area contain critical grading information, likely reflecting subtle tumor infiltration. Model performance declined with larger peritumoral distances, possibly due to increased normal tissue dilution.

**Conclusion:**

The radiomics features of the intratumoral region and the peritumoral region within 5 mm can optimize the preoperative grading of gliomas, providing support for surgical planning and prognostic evaluation.

## 1 INTRODUCTION

Cerebral glioma is the most common malignant brain tumor, accounting for approximately 78% of cases [1]. It is characterized by high invasiveness, high mortality, and frequent recurrence [2]. Adult-type diffuse gliomas are the most common subtype of cerebral gliomas [3]. Glioblastomas, which comprise approximately 54% of high-grade gliomas in adults, are associated with reduced survival rates with increasing age, dropping from 27% to as low as 3% [1].

The 2021 World Health Organization (WHO) Central Nervous System (CNS) (WHO CNS) tumor classification encompasses advancements in molecular mechanics and histopathology, enabling personalized treatment approaches that improve patient prognosis [4]. This classification categorizes adult-type diffuse gliomas into three types and grades (grades 2–4). The conventional treatment approach involves maximum tumor removal while preserving the patient's function. If the tumor is not in a functional area, the resection margin has to be expanded by 1–2 cm. According to this treatment strategy, patients with low-grade gliomas generally have a relatively good prognosis. However, for high-grade gliomas, particularly newly diagnosed grade 4 glioblastomas, their significant invasive ability means that even with a resection range between 95% and 100%, survival time is extended with an increase in resection rate [5]. These findings highlight the critical importance of accurately defining the actual boundaries of the tumor [6, 7].

Emerging evidence suggests that glioma heterogeneity extends beyond the tumor, influencing the surrounding regions and contributing to high recurrence rates [8]. Research has primarily focused on the peritumoral edema region [9, 10]. However, some biopsy studies have depicted that tumor cell infiltration extends beyond this area, even with regions without apparent signal changes on conventional magnetic resonance imaging (MRI) [11]. The areas beyond the peritumoral edema may harbor significant biological information, warranting further investigation to refine glioma diagnosis, grading, and treatment strategies.

Radiomics, a technique that extracts quantitative features from medical images and correlates them with tumor biological behavior, has demonstrated promise in the early diagnosis, grading, classification, treatment evaluation, and prognosis prediction of gliomas. Studies have indicated that radiomics models combining the tumor body (including both enhanced and non-enhanced areas within the tumor) with the peritumoral edema region offer superior preoperative grading prediction efficiency compared to those based solely on the enhanced, unenhanced, or peritumoral edema areas within the tumor [12, 13]. However, recent research has primarily focused on the tumor body and the peritumoral edema area, while regions beyond the peritumoral edema are yet to be integrated into radiomics-based models to aid in the early diagnosis and grading of gliomas.

This study explores whether the radiomics features of areas outside peritumoral edema can provide valuable information for preoperative, non-invasive glioma grading based on the 2021 WHO CNS tumor classification standards. The study seeks to enhance grading prediction accuracy using non-invasive radiomics methods and ultimately assists in developing precise, personalized treatment plans for clinical use.

## 2 MATERIALS AND METHODS

### 2.1 Patients

The University of California, San Francisco Preoperative Diffuse Glioma MRI (UCSF-PDGM) dataset [14] from The Cancer Imaging Archive (TCIA) [15] includes preoperative multiparametric MRI (mpMRI) scans of 180 patients with adult-type diffuse glioma. This dataset comprises 90 cases of grade 2 and 3 gliomas (51 cases of grade 2 and 39 of grade 3) and 90 cases of grade 4 gliomas. Samples with poor image quality were excluded during the selection process. Information on gender, age, and tumor grade according to the 2021 WHO CNS classification was retained for the included samples.

The included images comprised axial T1-weighted imaging (T1WI), T2-weighted imaging (T2WI), T2 fluid-attenuated inversion-recovery imaging (T2-FLAIR), diffusion-weighted imaging (DWI), and apparent diffusion coefficient (ADC). All preoperative mpMRI images were pre-registered, resampled (1 × 1 × 1 mm^3^), and underwent skull stripping.

A total of 180 patients were randomly divided into training and validation cohorts in a 7:3 ratio.

### 2.2 Tumor Segmentation

MRI images were reviewed, and tumor regions were delineated by two radiologists, one with three years of experience and the other with over five years of experience in diagnosing CNS tumors. Both radiologists were blinded to the grading results. They used an open-source software (ITK-SNAP, version 3.8.0, http://www.itksnap.org/) [16] to manually delineate the regions of interest (Volume of Interest, VOI) on T2WI and T2-FLAIR sequences, followed by copying the VOI to other sequences. The intratumoral VOI (VOI_I) encompassed the entire tumor body and peritumoral edema without excluding areas of cystic degeneration or necrosis within the tumor. Next, we utilized the Python 'SimpleITK' library to automatically expand the VOI at various distances (1, 2, 3, 4, 5, 10, and 15 mm), eliminating the original VOI_I to form a band-like peritumoral region. Fig. (**1**) demonstrates the tumor segmentation process along with its expansion into the adjacent peritumoral region. In summary, three distinct regional categories were established: (1) intratumoral regions only (VOI_I); (2) peritumoral regions only (VOI_P), comprising VOI_P1 to VOI_P5, VOI_P10, and VOI_P15; and (3) a combined category integrating both intratumoral and peritumoral regions, namely VOI_1 to VOI_5, VOI_10, and VOI_15.

### 2.3 Feature Extraction

Radiomics features were extracted from the aforementioned VOIs using the Pyradiomics package in Python (version 3.7.9), following the international Biomarker Standardization Initiative guidelines. Radiomics feature extraction was performed on the T1WI, T2WI, T2-FLAIR, DWI, and ADC data, which were manually segmented for each VOI. A total of 107 radiomics features were extracted for subsequent radiomics analysis for each patient and each sequence. The extracted features included first-order statistics, shape features, Gray Level Co-occurrence Matrix (GLCM), Gray Level Dependence Matrix (GLDM), Gray Level Run Length Matrix (GLRLM), Gray Level Zone Size Matrix (GLZSM), and Neighboring Gray Tone Difference Matrix (NGTDM) [17].

### 2.4 Statistical Analysis and Model Evaluation

Using the Statistical Package for the Social Sciences software (SPSS, version 26.0; IBM Corp, Armonk, USA), chi-square and Mann–Whitney U tests were performed to evaluate the gender and age differences between patients with grade 2/3 and grade 4 glioma within the training and validation cohorts, as well as the overall gender and age differences between the training and validation cohorts. A two-tailed p-value < 0.05 was considered statistically significant. Python software was used to calculate the area under the curve (AUC), accuracy, sensitivity, specificity, and F1 score to assess the quantitative discrimination performance of radiomic features in training and validation cohorts. The DeLong test was utilized to compare the AUC values across various models, with a significance level indicated by *p < 0.05*.

### 2.5 Feature Selection

The T2-FLAIR data of 40 randomly selected patients were re-evaluated by the two radiologists who previously delineated the VOIs under blinded settings for VOI re-delineation and radiomics feature extraction to measure inter-observer repeatability. To examine intra-observer repeatability, each radiologist performed the radiomics feature extraction twice within a week, following the same procedure. The intraclass correlation coefficient (ICC) was used to assess feature extraction consistency for both inter- and intra-observer agreement.

To minimize overfitting and potential bias in our radiomics model, only features with an ICC greater than 0.80 were retained [18]. The subsequent feature selection steps were designed to progressively refine the feature set based on three criteria: statistical significance, redundancy, and model relevance. First, Levene's test and t-test were applied to retain features with significant group differences (p < 0.05), ensuring initial exclusion of non-discriminatory features. Second, the mRMR algorithm was used to eliminate redundant features by maximizing feature-class relevance while minimizing inter-feature correlation, addressing the 'curse of dimensionality' in high-dimensional radiomics data. Finally, LASSO regression with cross-validation was employed to select features with non-zero coefficients, optimizing model sparsity and preventing overfitting. This hierarchical approach ensures that features are statistically meaningful, non-redundant, and algorithm-specifically relevant. Adjusting the order (*e.g*., applying LASSO before statistical filtering) could retain noisy or irrelevant features, while using a single method (*e.g*., only LASSO) might fail to sufficiently reduce redundancy, compromising model stability and interpretability. Subsequently, three feature selection steps were applied and performed exclusively on the training cohort.

First, the data were compared between the two groups using Levene's test and t-test, with statistically significant differences (*p* < 0.05) identified. Correlation analysis was then used to exclude characteristics with correlation coefficients larger than 0.9, reducing data redundancy and preventing multicollinearity issues. Second, the minimum redundancy maximum relevance (mRMR) algorithm was used to select the top 10 features, preventing overfitting caused by the “curse of dimensionality” due to an excessive number of features compared to the sample size [19]. Third, the least absolute shrinkage and selection operator (LASSO) was applied in feature selection, with tenfold cross-validation used to determine the optimal value for the parameter λ. All features with non-zero coefficients were retained, and the relevant content is illustrated in Supplementary File (Figs. **S1**-**S3**). Specifically, Fig. (**S1**) presents the feature selection process based on the least absolute shrinkage and selection operator (LASSO) method, which involves identifying the optimal parameter λ in the LASSO model using tenfold cross-validation. The retention of all features with non-zero coefficients using the LASSO method is shown in Fig. (**S2**). Additionally, Fig. (**S3**) displays the final selected radiomic features and their importance in each VOI. For all three figures, (a) corresponds to intratumoral volumes (VOI_I), (b–h) to peritumoral volumes (VOI_P1–5, 10, 15), and (i–o) to the combined category integrating both intratumoral and peritumoral regions (VOI_1–5, 10, 15). All steps were performed using Python.

### 2.6 Establishment of the Radiomics Model

We built the final LASSO regression model with the specified radiomics features and generated the radiomics score for each patient. We developed three distinct radiomics models: (1) the VOI_I model, based solely on intratumoral radiomics; (2) the VOI_P model, centered on peritumoral radiomics; (3) the combined radiomics model, which includes both intratumoral and peritumoral radiomics features.

### 2.7 Establishment of the Combined Clinical-radiomics Model

The clinical features that expressed statistically significant differences within the training and validation cohorts, or between the cohorts, were retained and combined with the radiomics scores to construct the clinical-radiomics combined model (combined-clinical model).

## 3 RESULTS

### 3.1 Epidemiological Characteristics

Table **1** presents the clinical features of the patients. No significant differences were observed in age (*p = 0.810*) or gender (*p = 0.131*) between the training and validation cohorts. However, significant differences were found in age between patients with grade 2/3 and grade 4 glioma within both the training and validation cohorts (*p* < 0.001 for both cohorts), while no significant differences were observed in gender (*p* > 0.05 for both cohorts).

### 3.2 Performance Assessment of the VOI_I Model and Combined Radiomics Models

**Fig. (2):**
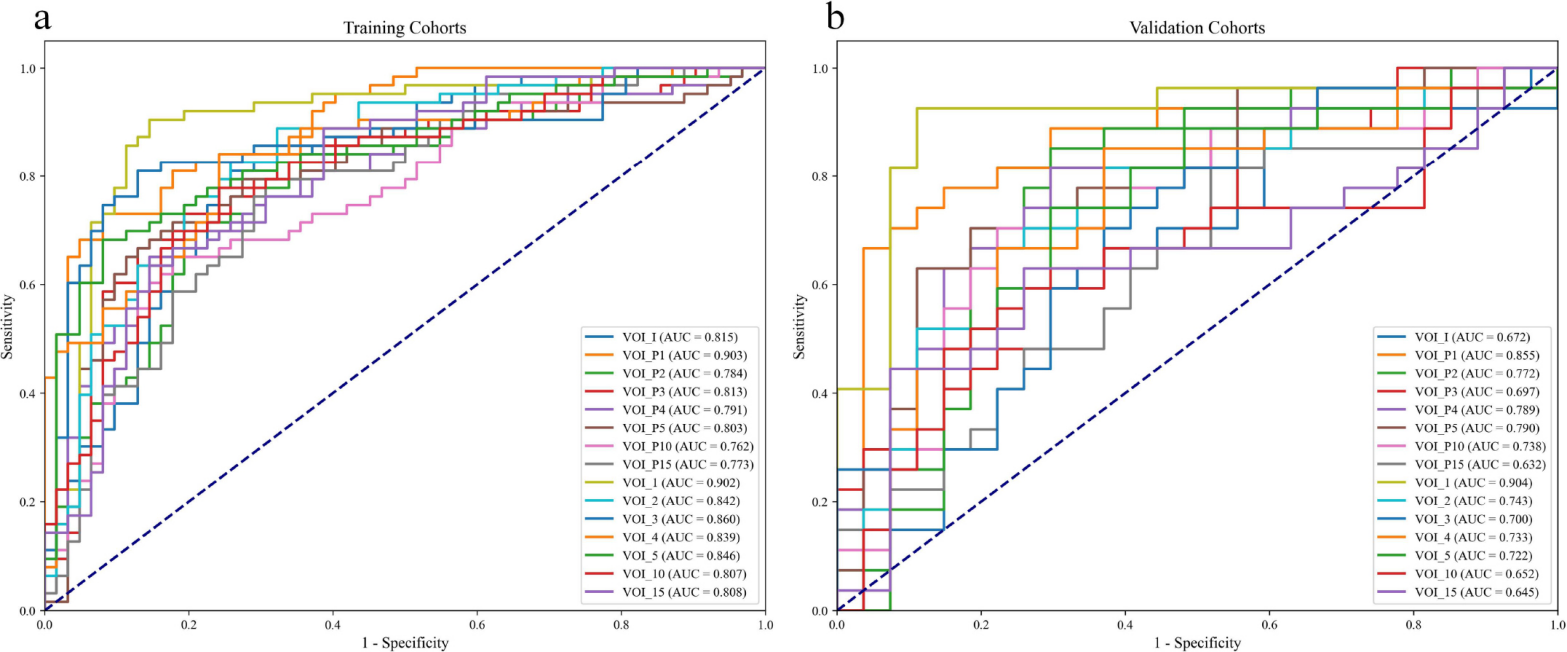
ROC curves and AUC values for each model in the training cohort (**a**) and validation cohort (**b**). **Note:** AUC—Area Under the Curve; ROC—Receiver Operating Characteristic.

In the training cohort, the AUC of the VOI_I model was 0.815 (95% confidence interval (CI): 0.742–0.883), with accuracy, sensitivity, specificity, and F1 scores of 0.768, 0.794, 0.742, and 0.775, respectively. The AUC of the VOI_1–5 models was higher than that of the VOI_I model in both the training and validation cohorts, with the best performance observed in the VOI_1 model, which achieved an AUC of 0.903 (95% CI: 0.844–0.957), with accuracy, sensitivity, specificity, and F1 score of 0.880, 0.905, 0.855, and 0.884, respectively. The VOI_1 model also performed best in the validation cohort, with an AUC of 0.904 (95% CI: 0.804–0.983), accuracy of 0.852, sensitivity of 0.778, specificity of 0.926, and an F1 score of 0.840. Detailed results are depicted in Tables **2** and **3**. The ROC curves and AUC values for each model in the training cohort (Fig. **2a**) and validation cohort (Fig. **2b**) are presented in Fig. (**2**). Notably, while the VOI_1 model demonstrated consistent AUC performance (0.903 *vs*. 0.904) across training and validation cohorts, minor discrepancies were observed in sensitivity (0.903 *vs*. 0.778) and specificity (0.855 *vs*. 0.926). These differences may reflect sampling variability inherent to retrospective cohort splitting and the smaller sample size of the validation cohort (n=54), which can amplify statistical noise. Further validation in larger, independent datasets is warranted to address this. The feature weights retained by VOI_1 are presented in Fig. (**3**), and the specific features retained by each VOI, along with their relevant importance, are displayed in Supplementary File (Figs. **S2** and **S3**).

**Fig. (4):**
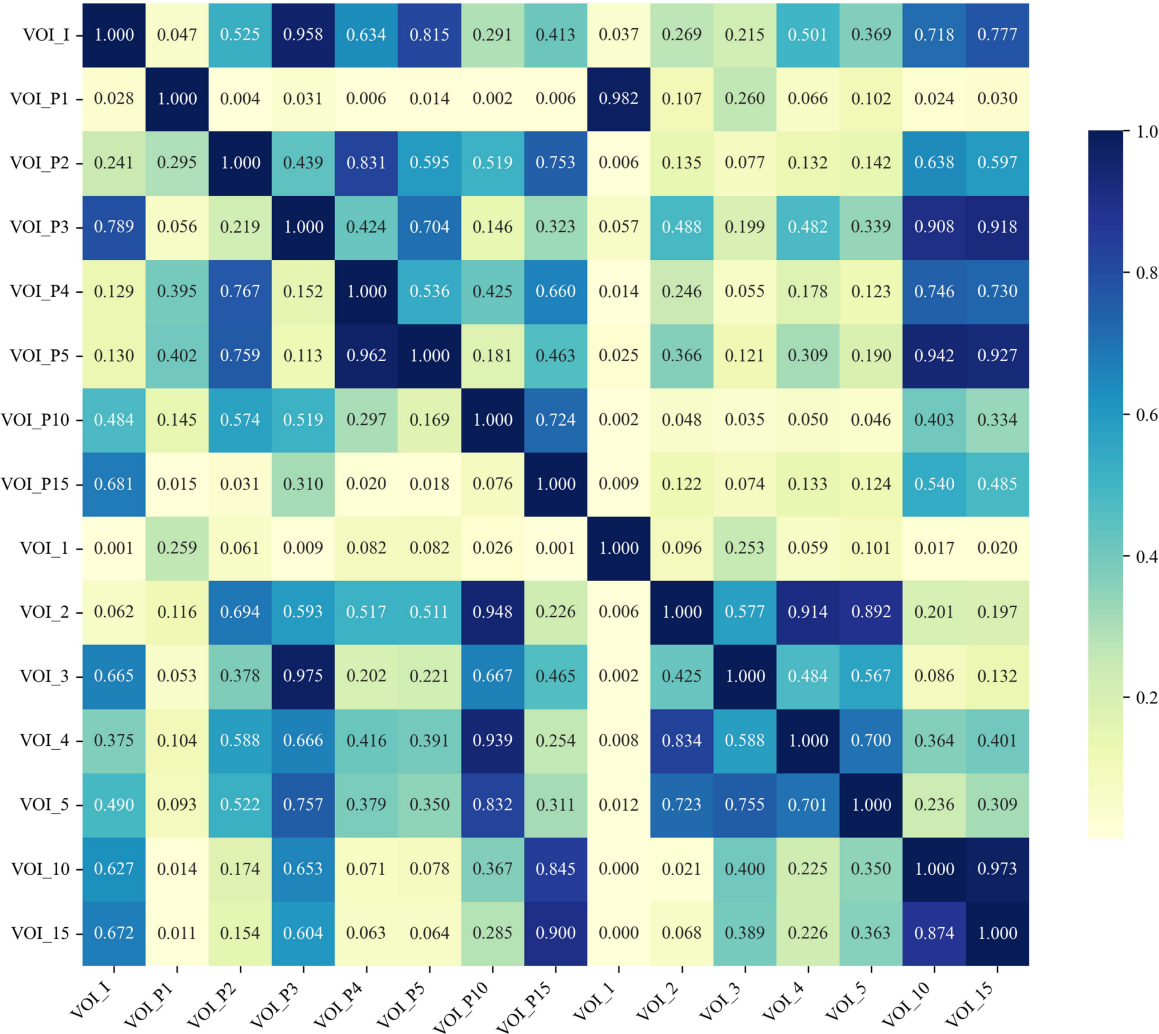
The upper triangular region shows the statistical significance of performance differences between models in the training cohort, while the lower triangular region shows the significance in the validation cohort.

The DeLong test was applied to assess AUC variations between the models across both the training and validation cohorts (Fig. **4**). The VOI_1 model demonstrated significantly better predictive performance than the VOI_I model, with *p*-values of 0.037 and 0.002 in training and validation cohorts, respectively.

### 3.3 Performance Assessment of the VOI_P Models

The VOI_P1 model, which focuses on the 1 mm peritu-moral region, demonstrated superior performance compared to other VOI_P models in both the training and validation cohorts. It achieved AUC values of 0.903 (accuracy, 0.816; sensitivity, 0.810; specificity, 0.823; F1, 0.816) and 0.855 (accuracy, 0.796; sensitivity, 0.741; specificity, 0.852; F1, 0.784), respectively. Detailed results are illustrated in Table **3**.

### 3.4 Predictive Performance of the Combined Clinical Model

The radiomics score for each patient in the VOI_1 model was determined and combined with the clinical feature of age to create a combined clinical-radiomics model (combined-clinical model). The performance of this combined model was examined in both cohorts. In the training cohort, the combined model achieved an AUC of 0.908 (95% CI: 0.851–0.959), with accuracy, sensitivity, specificity, and F1 score values of 0.872, 0.889, 0.855, and 0.875, respectively. In the validation cohort, AUC was 0.920 (95% CI: 0.828–0.989), with accuracy, sensitivity, specificity, and F1 score values of 0.870, 0.815, 0.926, and 0.863, respectively.

**Fig. (5):**
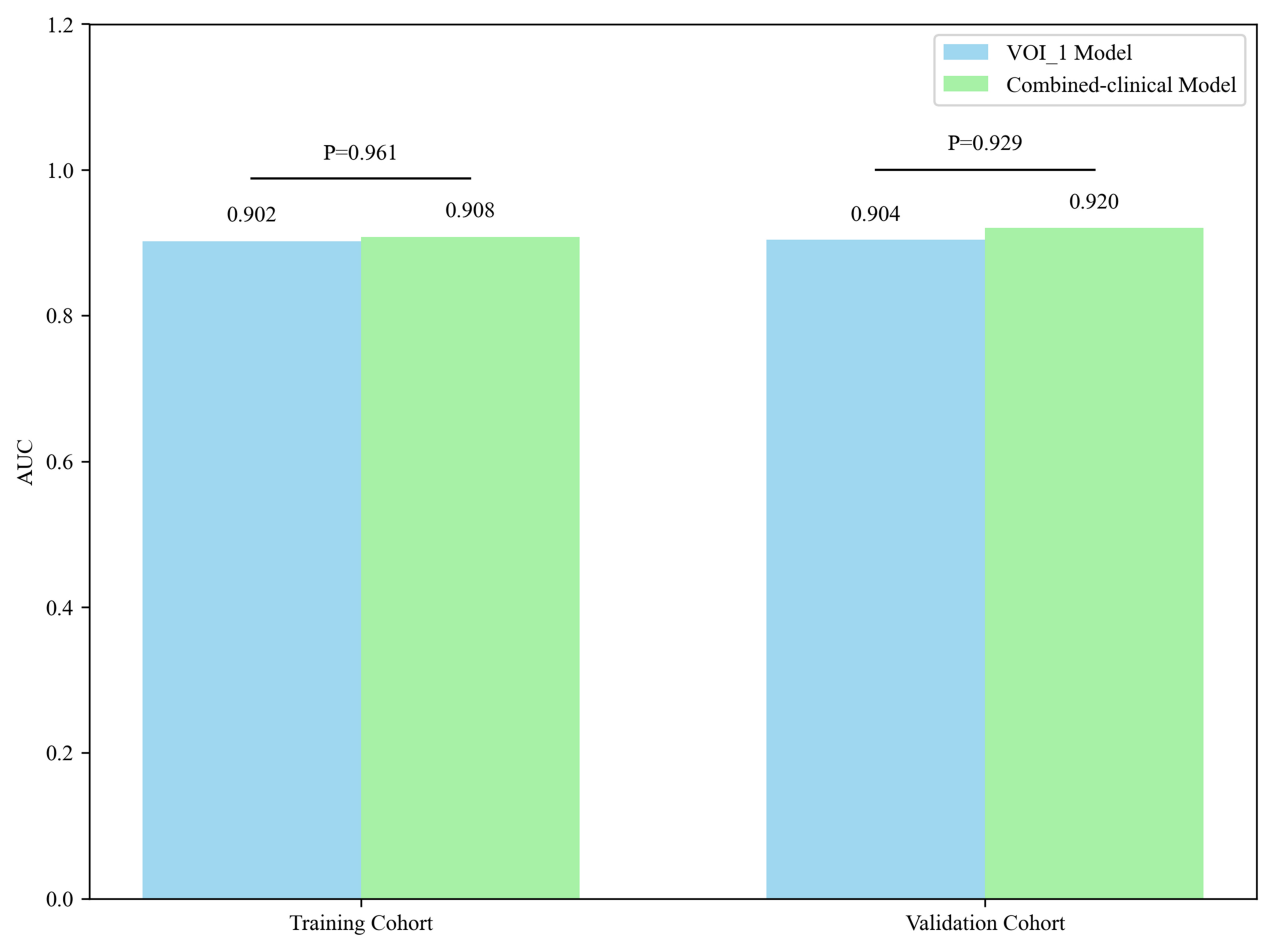
The AUC variations between the VOI_1 and combined-clinical models in the training and validation cohorts. The VOI_1 model is shown in blue, and the combined-clinical model in green.

Next, DeLong’s test was used to compare the AUC differences between the VOI_1 model and the combined-clinical model within both cohorts (Fig. **5**). The results demonstrated that there was no significant difference in the predictive performance between the VOI_1 model and the combined-clinical model, with p-values of 0.961 and 0.929 for training and validation cohorts, respectively.

## 4 DISCUSSION

### 4.1 Key Findings

Accurate grading of adult-type diffuse gliomas is critical for developing personalized treatment plans. This study developed three kinds of radiomics models: intratumoral (VOI_I), peritumoral (VOI_P), and a combined intratumoral-peritumoral model. The results revealed that the combined intratumoral-peritumoral model has great potential in preoperatively predicting the grade of adult-type diffuse glioma. The results showed that the VOI1-5 models have great potential in preoperatively predicting the grade of adult-type diffuse gliomas, among which the combined model of the intratumoral and 1mm peritumoral regions demonstrated the best predictive performance, significantly outperforming the separate intratumoral or peritumoral models. However, slight differences were observed in sensitivity (0.905 *vs*. 0.778) and specificity (0.855 *vs*. 0.926) between the training and validation cohorts for the VOI_1 model. This discrepancy may stem from two primary factors: 1) sampling variation due to the retrospective random split, where the validation cohort might contain more heterogeneous cases (*e.g*., tumors with atypical infiltration patterns); and 2) the smaller sample size of the validation cohort (n=54), which increases susceptibility to statistical noise. Notably, the stable AUC across cohorts (0.903 *vs.* 0.904) indicates consistent overall discriminative power. However, there was no significant difference in performance compared to the clinical-radiomics model (*p* > 0.05). Although LASSO regression was used here for its interpretability and feature selection efficiency, deep learning approaches have shown potential in medical image analysis for capturing hierarchical patterns in multi-sequence MRI [20, 21]. These methods could enhance the detection of subtle tumor-infiltrative features in peritumoral regions. While this study focused on demonstrating the value of peritumoral radiomics using traditional machine learning, future work may explore deep learning-based models to further improve grading accuracy by leveraging automated feature learning from complex imaging data.

### 4.2 Comparison with Existing Studies

Research on the additional relevance of radiomic features from peritumoral regions beyond peritumoral edema areas in the preoperative grading of adult-type diffuse gliomas remains limited. For instance, Zhou *et al*. [22] only investigated the radiomic features of the tumor body in the preoperative non-invasive grading of gliomas. Similarly, studies by van der Voort *et al*. [23] and Lin *et al*. [24] focused solely on the tumor body and peritumoral edema. Based on the 2016 WHO CNS tumor classification, Cheng *et al*. [25], investigated the role of radiomic features in distinguishing grade I/II from grade III/IV gliomas. In their study, the peritumoral region was defined as extending 1–5 mm from the tumor body (including both enhancing and non-enhancing regions), which overlapped with the peritumoral edema. The results indicated that the prediction model incorporating both tumor body and peritumoral radiomic features exhibited high predictive performance, particularly in the peritumoral 1 mm region, where the AUC was 0.968. However, the study did not investigate the independent significance of the regions beyond the peritumoral edema, where no signal changes are detected by conventional MRI sequences in the preoperative prediction of glioma grading.

### 4.3 Methodological Improvements and Insights

This study further improved this framework by defining the tumor body and peritumoral edema as the “intratumoral regions” and extending the research range to 15 mm. We defined seven different peritumoral region ranges (1–5, 10, and 15 mm), all located beyond the peritumoral edema in areas where conventional MRI sequences detect no signal changes. This approach aimed to explore the independent value of these regions in the preoperative grading of gliomas. Notably, while this study integrated features from multiple MRI sequences (T1WI T2WI T2-FLAIR DWI ADC) the specific contribution of functional sequences like DWI/ADC—known to reflect cellular density and water diffusion in gliomas—was not explicitly evaluated. DWI-derived parameters such as apparent diffusion coefficient (ADC) have been shown in prior studies to correlate with tumor cellularity and grading, suggesting their critical role in differentiating infiltrative margins. Future studies should employ techniques like SHAP (SHapley Additive exPlanations) values or feature weight analysis to quantify the contribution of each sequence, ensuring functional imaging information is optimally leveraged. Additionally developing multimodal fusion models that combine structural sequences (*e.g*., T2-FLAIR) with functional data (*e.g*., DWI) could enhance the model’s ability to distinguish between tumor infiltration and reactive edema, potentially improving specificity for high-grade gliomas by integrating complementary structural and functional insights into tumor biology.

The findings of this study demonstrate that the combined radiomics model, which includes both the intratumoral and peritumoral 1 mm regions (VOI_1), exhibits significantly superior predictive performance in glioma grading than the intratumoral-only model (VOI_I). This demonstration implies that regions beyond peritumoral edema provide important predictive information correlating with glioma grading. These findings are consistent with those of Zetterling *et al*. [11], who compared MRI data with biopsy samples obtained from gliomas undergoing gross total resection, revealing that tumor infiltration extends beyond regions with abnormal MRI signals. Clinically, the challenge of tumor cell infiltration beyond radiological boundaries has prompted several therapeutic explorations. For instance, neurosurgeons often adopt an extended resection margin (1–2 cm beyond visible tumor margins) for high-grade gliomas to reduce microscopic residual disease, particularly in non-functional brain areas. Intraoperative techniques such as 5-aminolevulinic acid (5-ALA) fluorescence guidance and frozen section pathology are used to identify infiltrative margins, though these remain invasive and time-consuming. Systemic therapies targeting tumor microenvironment—such as anti-angiogenic agents (*e.g*., bevacizumab)—may also address occult infiltration by disrupting neovascularization in infiltrative zones. Our findings suggest that radiomics features from the 1-mm peritumoral region could preoperatively stratify patients into high-risk (needing aggressive resection or adjuvant therapy) and low-risk groups, potentially optimizing treatment personalization. Specifically, the VOI_1 model’s predictive power for tumor infiltration beyond visible edema can inform surgical planning by guiding neurosurgeons to consider extending resection margins to 1.5–2 cm beyond the T2-FLAIR hyperintense boundary for high VOI_1 score patients (indicating aggressive infiltration) in line with clinical practices for high-grade gliomas where broader margins correlate with improved survival while suggesting more conservative margins (0.5–1 cm) for low-scoring patients to reduce neurological deficit risks in functional areas, and can serve as a preoperative risk stratification tool to prioritize intraoperative frozen section analysis or 5-ALA fluorescence imaging in high-risk regions identified by VOI_1 features to enhance microscopic residual tumor detection as supported by studies showing radiomics-derived infiltration risk correlates with postoperative recurrence patterns despite the study’s lack of direct surgical outcome data and warranting future prospective validation through integration with intraoperative imaging like MRI-guided resection. Additionally, the model may inform adjuvant therapy strategies: high VOI_1 scores could indicate patients requiring intensified chemoradiation (*e.g*., temozolomide with radiotherapy) or targeted therapies (*e.g*., anti-angiogenic agents), while low-scoring patients might be candidates for more conservative post-resection monitoring. This approach aligns with the 2021 WHO classification’s emphasis on integrating molecular and imaging data for personalized glioma management, though validation in prospective trials is needed to establish clinical utility. Adult-type diffuse gliomas, particularly grade 4 gliomas, are marked by highly invasive behavior, characterized by tumor cell migration into adjacent brain tissue, disruption of normal structures, and induction of peritumoral morphological and microstructural changes. These slight changes, however, are often difficult to detect using conventional medical imaging. Radiomics can capture pathophysiological changes in the tumor microenvironment by extracting quantitative features from MRI images that are invisible to the naked eye [26]. This mechanism may be an important factor contributing to the superior predictive performance of the combined intratumoral-peritumoral radiomics model compared to the VOI_I model in this study.

Moreover, our findings demonstrate that radiomic features derived from the regions beyond the peritumoral edema alone are crucial in predicting the grading of adult-type diffuse gliomas. Among the VOI_P models, the VOI_P1-based model achieved the highest performance in both training and validation cohorts, with AUC values of 0.903 (accuracy, 0.816; sensitivity, 0.810; specificity, 0.823; F1, 0.816) and 0.855 (accuracy, 0.796; sensitivity, 0.741; specificity, 0.852; F1, 0.784), respectively. This finding is consistent with the research by Wu *et al*. [27] on non-small cell lung cancer, which discovered that as the peritumoral region expands, the predictive performance of the model decreases, owing to an increase in the proportion of normal tissue and a relative decrease in tumor tissue within the extended area. Although our study focused on gliomas rather than lung cancer, our findings are significant. This hypothesis was partially validated in our study. We found that the radiomics models utilizing the 10 and 15 mm peritumoral regions (VOI_P10 and VOI_P15) had notably worse predictive performance compared to the VOI_P1 model in the training cohort. This finding is likely because 10 and 15 mm peritumoral regions are located further from the tumor body and include a higher proportion of normal brain tissue, making them less relevant for radiomic analysis. Similarly, in the combined intratumoral-peritumoral model, VOI_1 demonstrated significantly better predictive performance than VOI_10 and VOI_15 (*p-*values < 0.05).

While we observed that the VOI_1 model exhibited the best predictive performance, we also observed that its AUC was higher than that of the combined intratumoral-peritumoral radiomics models with peritumoral regions of 2–5 mm, both in training and validation cohorts. However, no significant differences were observed among these models in the training cohort (p > 0.05 for all comparisons). We believe that this finding is likely attributed to the complexity and biological variability of the tumor microenvironment. The microenvironment of diffuse gliomas includes features such as the invasiveness of tumor cells and angiogenesis, and immune responses. These factors likely result in high heterogeneity of radiomic features as the peritumoral region expands, thereby compromising the models’ stability and predictive performance. Furthermore, constraints in image data processing methods could affect the performance of radiomic models. For instance, calibration errors or inconsistencies in delineating tumor and peritumoral regions may exist. These factors can reduce the stability of the radiomic features, thereby impacting the model's predictive power.

### 4.4 Clinical Implications

However, the lack of statistically significant differences between models in the 1–5 mm peritumoral regions may be attributed to the relatively high stability and reproducibility of some radiomic properties in the peritumoral regions. When numerous radiomic models are constructed within a small range, their performance tends to be relatively stable. Although no studies have yet explored the reproducibility and repeatability of glioma peritumoral radiomic features, Tunali *et al*. [28] found that peritumoral CT radiomic features in lung cancer are stable and repeatable, implying these phenomena. These data suggest that future studies should comprehensively explore information within the 5 mm peritumoral region to improve the radiomic predictive models for adult-type diffuse gliomas and increase their clinical reliability.

### 4.5 Limitations and Future Directions

This study has several limitations. First, the sample size was limited, and the data was mainly derived from the UCSF-PDGM dataset in the TCIA public database, resulting in a homogeneous sample source. Second, only training and validation cohorts were used, with no independent external datasets for additional validation, which highlights the need for improved model robustness. Additionally, the exclusive use of the UCSF-PDGM dataset introduces two key limitations: 1) Imaging protocol homogeneity: All data were acquired at a single institution, potentially masking variability in radiomics features caused by differences in MRI scanners, pulse sequences, or reconstruction parameters. For example, T2-FLAIR signal intensity and texture features may differ significantly across devices, leading to inconsistent feature extraction. 2)Patient population bias: The dataset may not reflect the genetic and morphological heterogeneity of gliomas in diverse ethnic or geographic populations, which could affect model robustness. While preprocessing steps standardized image formats, they cannot fully account for inter-institutional variability. Third, the limited clinical features might account for the lack of a significant performance difference between the VOI_1 and combined clinical-radiomics models. Furthermore, the study’s reliance on a single training-validation cohort split (without independent external validation or repeated cross-validation) may introduce bias in assessing model generalizability. The random 7:3 dataset split, while following conventional radiomics practices, does not fully account for variability across imaging protocols or patient populations. This limitation is compounded by the homogeneous sample source (UCSF-PDGM dataset), which may not reflect real-world heterogeneity. Furthermore, the small sample size of the validation cohort (n=54) and reproducibility subset (n=40) may limit statistical power and introduce bias in assessing model stability. While the random 7:3 training-validation split followed standard practices, the validation cohort’s size is below the recommended threshold for robust radiomics validation (typically n≥100). This limitation is compounded by the absence of external validation, which is essential to confirm generalizability across diverse imaging protocols and patient populations. To mitigate these issues, future studies should: 1) expand the dataset using multi-center cohorts (*e.g*., CPTAC-Glioma, BraTS) to capture heterogeneity in tumor biology and imaging characteristics; 2) employ bootstrap resampling or repeated k-fold cross-validation to assess feature stability and reduce overfitting risks, as recommended by the Radiomics Quality Score (RQS) guidelines. These approaches will enhance confidence in the model’s robustness and facilitate its translation to clinical practice. Finally, the study lacks a direct correlation between radiomic features and histopathological infiltration patterns, as we did not perform 3D spatial registration of preoperative MRI with postoperative pathological specimens. This prevents quantitative assessment of how peritumoral radiomics features relate to tumor cell density, microvascular proliferation, or molecular markers (*e.g*., IDH mutation), 1p/19q codeletion—critical components of the 2021 WHO classification for gliomas. Future studies should employ digital pathology to co-register MRI with histology slides, enabling voxel-wise correlation analysis at 1–5 mm intervals from the tumor margin. Integrating intraoperative frozen section data—such as IDH mutation status or Ki-67 proliferation index—will also help validate whether radiomic features reflect biologically aggressive phenotypes, as recommended by the 2021 WHO classification for gliomas. Additionally, implementing peritumoral 1-mm radiomics analysis in clinical workflows faces practical challenges. Manual segmentation of the 1-mm peritumoral region requires high precision and is time-consuming, particularly in tumors with irregular margins, and while interclass correlation coefficients (ICC) exceeded 0.80, this may not fully account for interobserver variability in complex margins (*e.g*., subtle signal transitions), potentially introducing bias in feature extraction. Multicenter standardization of MRI sequences and preprocessing pipelines remains a hurdle, as differences in scanner protocols (*e.g*., T2-FLAIR parameters) can lead to inconsistent feature extraction across institutions. Furthermore, extracting and analyzing hundreds of radiomics features necessitates specialized software (*e.g*., Pyradiomics) and computational resources that may not be available in all clinical settings. Clinician familiarity with quantitative radiomics scores is another barrier, as integrating these metrics into routine practice would require training and validation across healthcare systems. Addressing these challenges will require the development of automated segmentation tools, the establishment of international imaging standards (*e.g*., following IBBSI guidelines), and collaborative efforts to bridge radiomics research with clinical workflows.

## CONCLUSION

This study used magnetic resonance radiomics to investigate the predictive significance of features from regions beyond peritumoral edema in the preoperative non-invasive grading of gliomas and developed radiomics models for several regional ranges. The study results indicate that radiomics features combining the intratumoral region and the peritumoral region within 1-5 mm are beneficial for improving the predictive efficacy of glioma grading. These findings provide valuable radiological information for glioma classification and offer objective guidance for clinical surgical planning, with significant implications for improving patient prognosis. For clinical implementation, the radiomics score could be incorporated into preoperative MRI reporting workflows as a supplementary grading indicator. Specifically, after standard MRI acquisition, radiologists would use open-source tools to automatically extract features from the tumor and the peritumoral region within 1-5 mm, generate a quantitative score, and integrate it with conventional imaging assessments to inform multidisciplinary treatment planning. This approach aligns with emerging standards for precision oncology, where radiomics complements histopathological and molecular diagnostics. While this study provides a proof-of-concept, prospective validation in multicenter clinical trials is essential to establish the model’s reliability across diverse imaging systems and patient populations. Collaboration with industry partners to develop regulatory-compliant tools will be critical for translating these findings into routine clinical practice.

## Figures and Tables

**Fig. (1):**
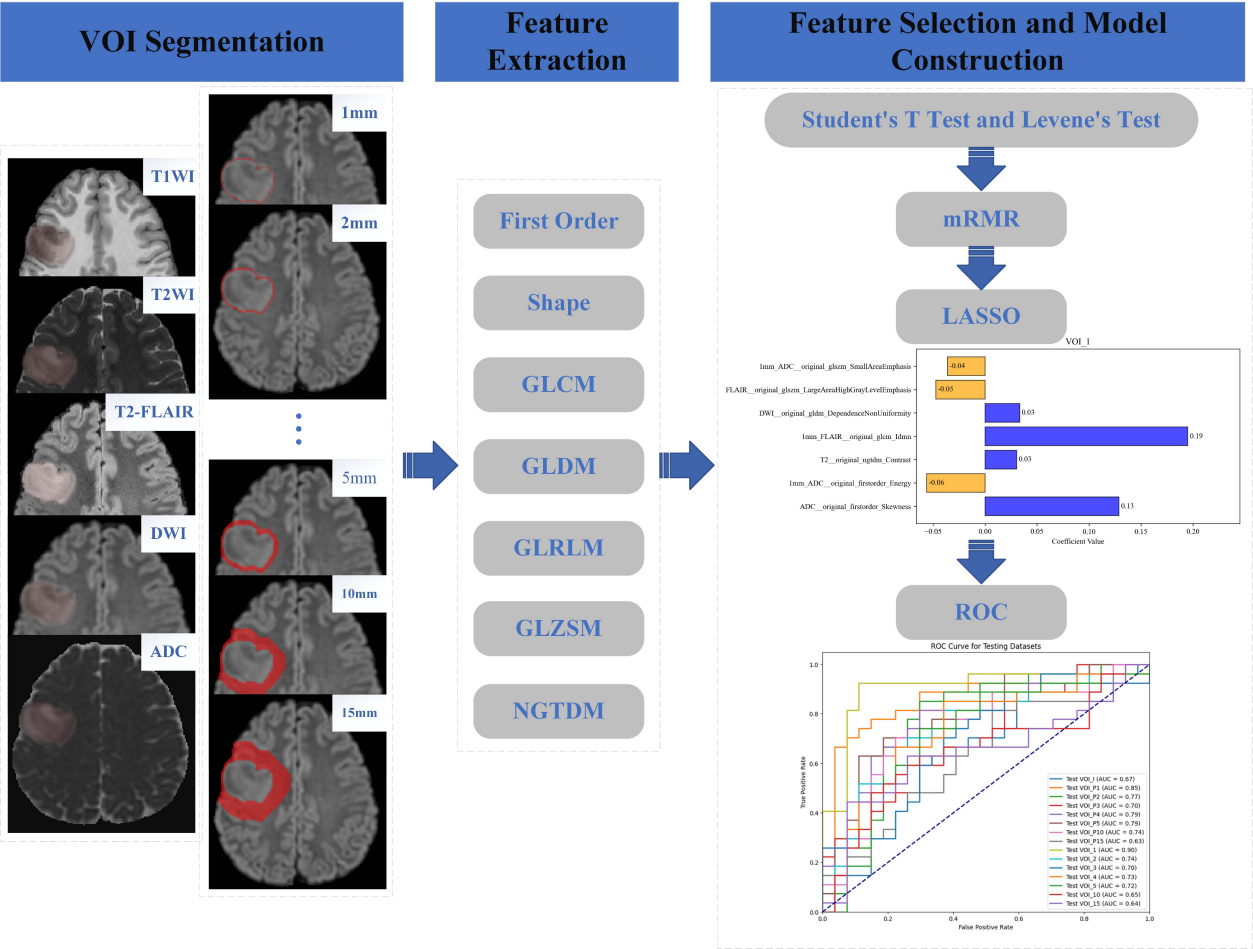
The workflow of the study. **Note:** VOI—volume of interest; mRMR—the minimum redundancy maximum relevance algorithm; LASSO—the least absolute shrinkage and selection operator.

**Fig. (3):**
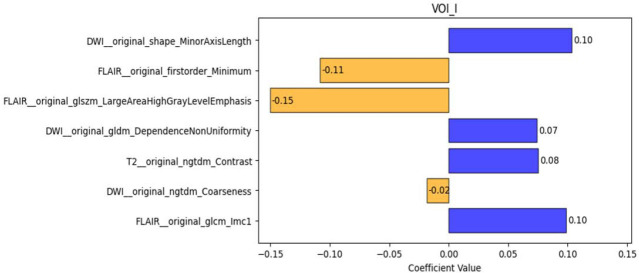
The features selected and their weights after feature selection in the VOI_1 model.

**Table 1:** Clinical characteristics of participants in the training and validation cohorts.

**Characteristic**	**Training Cohort**		**P**	**Validation Cohort**		**P**
	**Lower** **(n=63)**	**High** **(n=63)**		**Lower** **(n=27)**	**High** **(n=27)**	
Age, years, median	40(33,52)	60(54,68)	**<0.001**	36(28,48)	63(54,69)	**<0.001**
Gender (%)	-	-	**0.466**	-	-	**1.000**
Male	40(63.5)	36(57.1)	-	13(48.1)	13(48.1)	-
Female	23(36.5)	27(42.9)	-	14(51.9)	14(51.9)	-

**Table 2:** Performance assessment of intratumoral (VOI_I) and combined radiomics models.

**Model**		**TP(n)**	**TN(n)**	**FN(n)**	**FP(n)**	**AUC (95%CI)**	**Accuracy**	**Sensitivity**	**Specificity**	**F1**
**Training Cohorts**										
VOI_I	50		46	13	16	0.815(0.742 - 0.883)	0.768	0.794	0.742	0.775
**VOI_1**	57		53	6	9	**0.903(0.844 - 0.957)**	**0.880**	**0.905**	**0.855**	**0.884**
VOI_2	52		44	11	18	0.842(0.770 - 0.908)	0.768	0.825	0.710	0.782
VOI_3	52		51	11	11	0.860(0.785 - 0.923)	0.824	0.825	0.823	0.825
VOI_4	52		47	11	15	0.839(0.772 - 0.902)	0.792	0.825	0.758	0.800
VOI_5	49		48	14	14	0.846(0.770 - 0.907)	0.776	0.778	0.774	0.778
VOI_10	48		47	15	15	0.807(0.729 - 0.881)	0.760	0.762	0.758	0.762
VOI_15	49		39	14	23	0.808(0.726 - 0.879)	0.704	0.778	0.629	0.726
**Validation Cohorts**										
VOI_I	18		16	9	11	0.672(0.525 - 0.815)	0.630	0.667	0.593	0.643
**VOI_1**	21		25	6	2	**0.904(0.804 - 0.983)**	**0.852**	**0.778**	**0.926**	**0.840**
VOI_2	15		21	12	6	0.744(0.604 - 0.878)	0.667	0.556	0.778	0.625
VOI_3	15		19	12	8	0.700(0.544 - 0.835)	0.630	0.556	0.704	0.600
VOI_4	17		21	10	6	0.733(0.578 - 0.865)	0.704	0.630	0.778	0.680
VOI_5	20		17	7	10	0.722(0.570 - 0.859)	0.685	0.741	0.630	0.702
VOI_10	17		17	10	10	0.652(0.499 - 0.800)	0.630	0.630	0.630	0.630
VOI_15	17		20	10	7	0.645(0.484 - 0.798)	0.685	0.630	0.741	0.667

**Abbreviations:** TP = true positive, TN = true negative, FP = false positive, FN = false negative

**Table 3:** Performance assessment of peritumoral (VOI_I) models.

**Model**	**TP(n)**	**TN(n)**	**FN(n)**	**FP(n)**	**AUC (95%CI)**	**Accuracy**	**Sensitivity**	**Specificity**	**F1**
**Training Cohorts**									
VOI_P1	51	51	12	11	0.903(0.853 - 0.947)	0.816	0.810	0.823	0.816
VOI_P2	47	43	16	19	0.784(0.709 - 0.857)	0.720	0.746	0.694	0.729
VOI_P3	43	52	20	10	0.813(0.729 - 0.887)	0.760	0.683	0.839	0.741
VOI_P4	48	41	15	21	0.791(0.709 - 0.872)	0.712	0.762	0.661	0.727
VOI_P5	45	47	18	15	0.803(0.717 - 0.878)	0.736	0.714	0.758	0.732
VOI_P10	44	40	19	22	0.762(0.676 - 0.849)	0.672	0.698	0.645	0.682
VOI_P15	42	45	21	17	0.773(0.687 - 0.859)	0.696	0.667	0.726	0.689
**Validation Cohorts**									
VOI_P1	20	23	7	4	0.855(0.731 - 0.955)	0.796	0.741	0.852	0.784
VOI_P2	16	22	11	5	0.772(0.633 - 0.907)	0.704	0.593	0.815	0.667
VOI_P3	15	19	12	8	0.697(0.564 - 0.829)	0.630	0.556	0.704	0.600
VOI_P4	18	20	9	7	0.789(0.656 - 0.911)	0.704	0.667	0.741	0.692
VOI_P5	18	22	9	5	0.790(0.659 - 0.911)	0.741	0.667	0.815	0.720
VOI_P10	19	19	8	8	0.738(0.600 - 0.869)	0.704	0.704	0.704	0.704
VOI_P15	15	17	12	10	0.632(0.483 - 0.780)	0.593	0.556	0.630	0.577

**Abbreviations:** TP = true positive, TN = true negative, FP = false positive, FN = false negative.

## Data Availability

All data generated or analyzed during this study are included in this published article.

## LIST OF ABBREVIATIONS

MRI: Magnetic Resonance Imaging
VOI: Volume of Interest
VOI_I: the Intratumoral VOI
VOI_P: the Peritumoral VOI
AUC: Area Under the Curve
WHO: World Health Organization
CNS: Central Nervous System
UCSF-PDGM: University of California San Francisco Preoperative Diffuse Glioma MRI
TCIA: The Cancer Imaging Archive
mpMRI: Multiparametric Magnetic Resonance Imaging
T1WI: T1-weighted Imaging
T2WI: T2-weighted Imaging
T2-FLAIR: T2-Fluid Attenuated Inversion Recovery
DWI: Diffusion-weighted Imaging
ADC: Apparent Diffusion Coefficient
GLCM: Gray Level Co-occurrence Matrix
GLDM: Gray Level Dependence Matrix
GLRLM: Gray Level Run Length Matrix
GLSZM: Gray Level Size Zone Matrix
NGTDM: Neighboring Gray Tone Difference Matrix
ICC: Intraclass Correlation Coefficient
mRMR: Minimum Redundancy Maximum Relevance
LASSO: Least Absolute Shrinkage and Selection Operator
CI: Confidence Interval

## STANDARDS OF REPORTING

STROBE guidelines were followed.
