# Supplementary material for: Exploring the Predictive Value of Grading in Regions Beyond Peritumoral Edema in Gliomas based on Radiomics
Source: Curr Med Imaging. 2025 Aug 28;21:e15734056387494. doi: 10.2174/0115734056387494250823132119 (PMC13223437; doi:10.2174/0115734056387494250823132119)
Supplement: Supplementary file 1 [file CMIM-21-E15734056387494_SD1.pdf]

1573-4056/25 *Send Orders for Reprints to reprints@benthamscience.net*

BENTHAM SCIENCE

CrossMark

Current Medical Imaging

Content list available at: https://benthamscience.com/journals/cmir

Supplementary Material

Exploring the Predictive Value of Grading in Regions Beyond Peritumoral Edema in Gliomas Based on Radiomics

Jie Pan[1], Jun Lu[1], Shaohua Peng[1,*] and Minhai Wang[1]

[1]*Department of Medical Imaging Center, The First Affiliated Hospital of Medical College, Shihezi University, Medical Imaging Center, Shihezi, China*

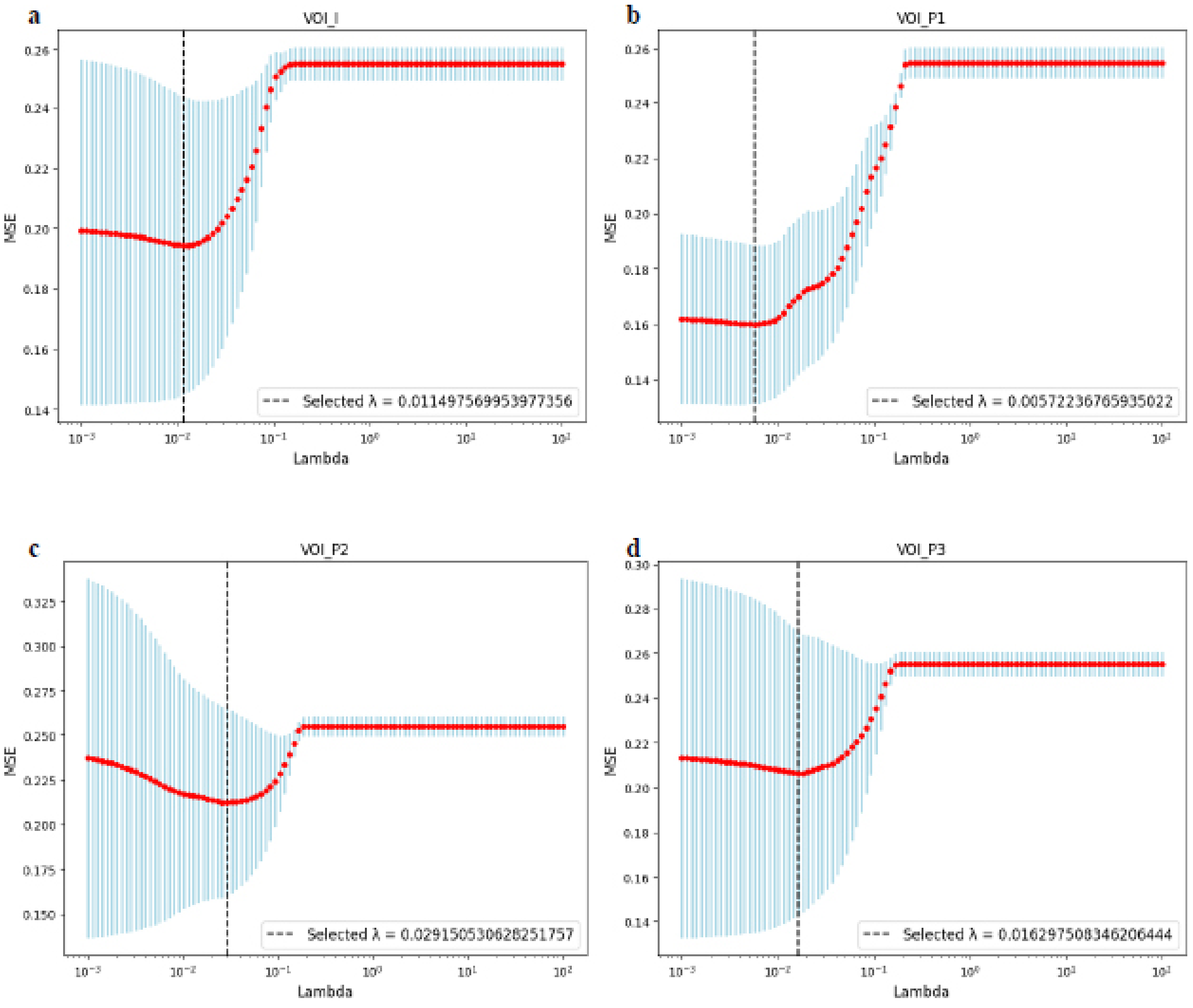

DOI: 10.2174/0115734056387494250823132119, 2025, *21*, e15734056387494

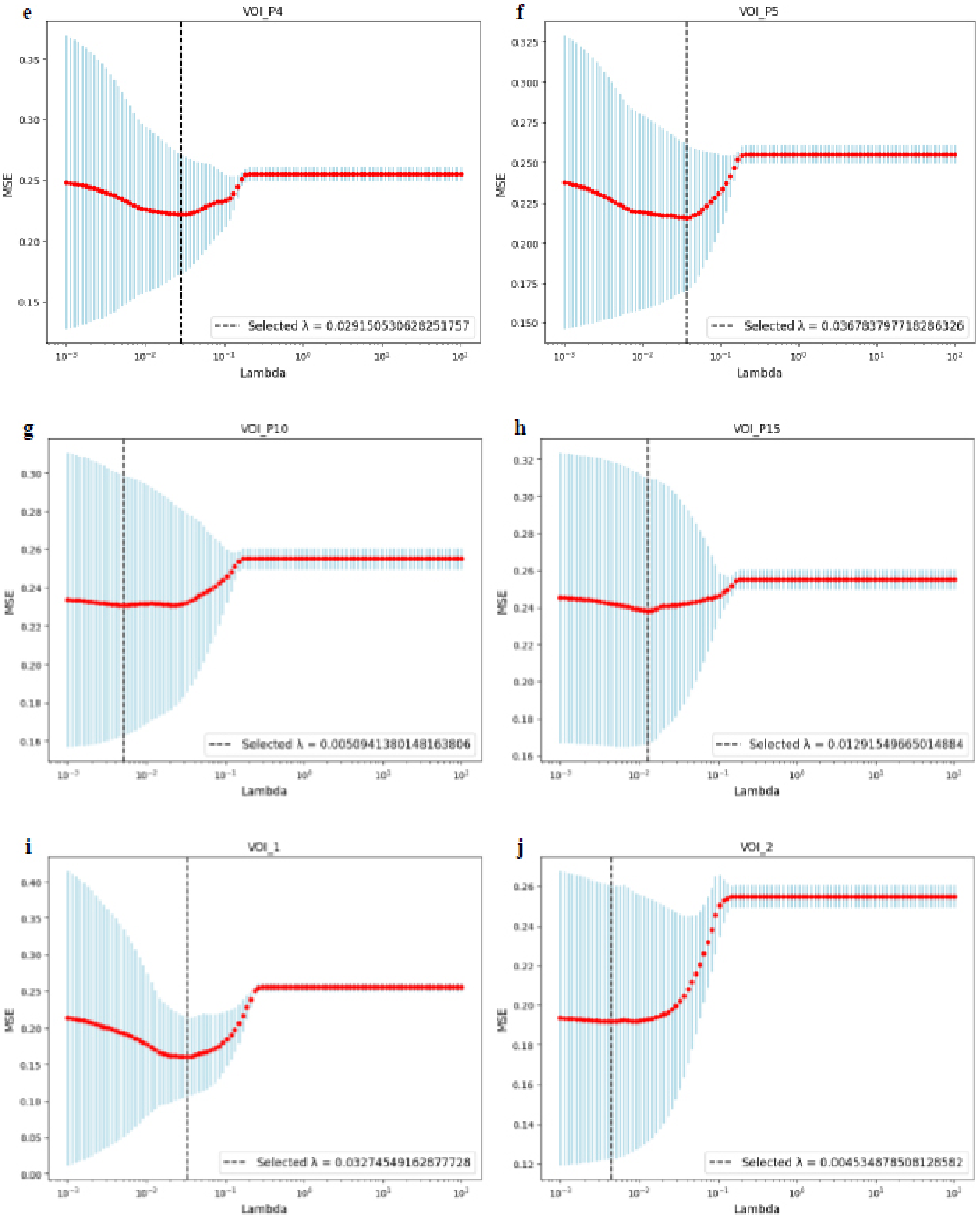
e
VOI_P4
MSE
Lambda
Selected λ = 0.029150530628251757
f
VOI_P5
MSE
Lambda
Selected λ = 0.036783797718286326
g
VOI_P10
MSE
Lambda
Selected λ = 0.0050941380148163806
h
VOI_P15
MSE
Lambda
Selected λ = 0.01291549665014884
i
VOI_1
MSE
Lambda
Selected λ = 0.03274549162877728
j
VOI_2
MSE
Lambda
Selected λ = 0.004534878508128582

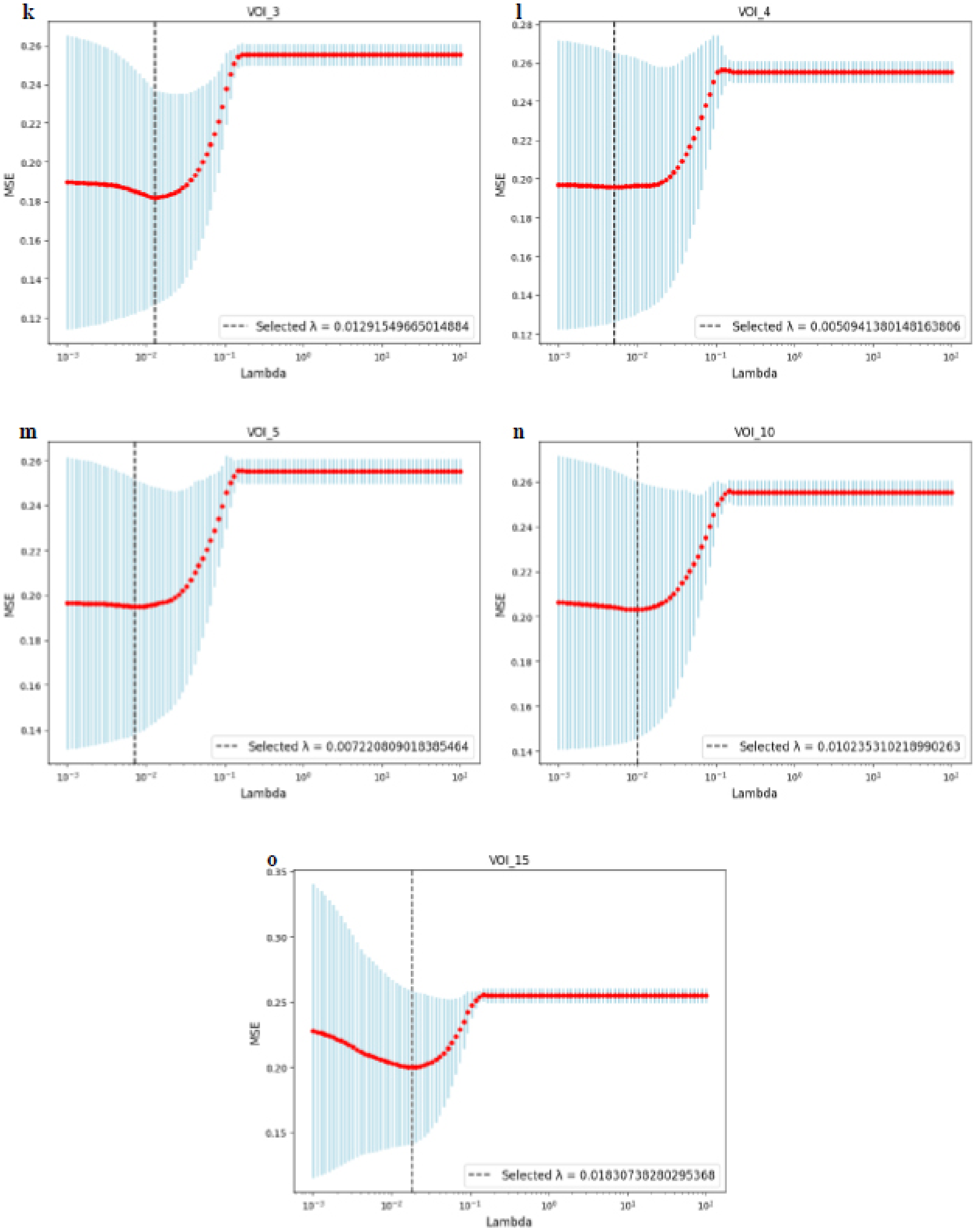

Fig. (S1). (**a**) shows the feature selection process for the features extracted from the intratumoral volumes (VOI_I, including tumor body and edema) using the Least Absolute Shrinkage and Selection Operator (LASSO) method. The optimal parameter λ in the LASSO model was determined via tenfold cross-validation, and vertical lines were drawn at the optimal values based on the minimum criteria. Peritumoral volumes (VOI_P): (**b**-**h**); combined category integrating both intratumoral and peritumoral regions: (**i**-**o**).

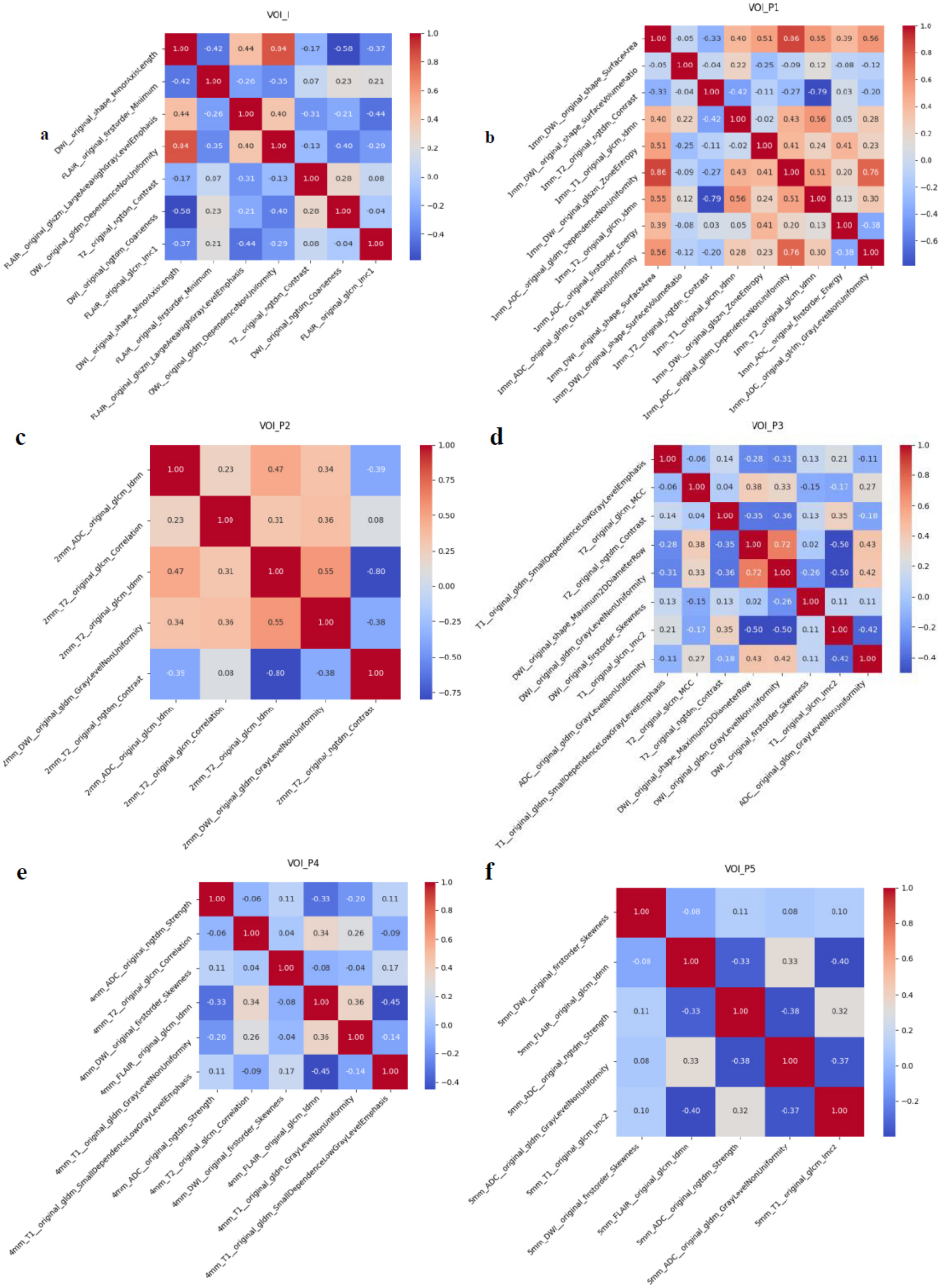
a
VOI_I
DWI__original_shape_MinorAxisLength
FLAIR__original_firstorder_Minimum
FLAIR__original_glszm_LargeAreaHighGrayLevelEmphasis
OWI__original_gldm_DependenceNonUniformity
T2__original_ngtdm_Contrast
DWI__original_ngtdm_Coarseness
FLAIR__original_glcm_Imc1
b
VOI_P1
1mm_DWI__original_shape_SurfaceArea
1mm_DWI__original_shape_SurfaceVolumeRatio
1mm_T2__original_ngtdm_Contrast
1mm_T1__original_glcm_Idmn
1mm_DWI__original_glszm_ZoneEntropy
1mm_ADC__original_gldm_DependenceNonUniformity
1mm_T2__original_glcm_Idmn
1mm_ADC__original_firstorder_Energy
1mm_ADC__original_glrlm_GrayLevelNonUniformity
c
VOI_P2
2mm_ADC__original_glcm_Idmn
2mm_T2__original_glcm_Correlation
2mm_T2__original_glcm_Idmn
2mm_DWI__original_gldm_GrayLevelNonUniformity
2mm_T2__original_ngtdm_Contrast
d
VOI_P3
T1__original_gldm_SmallDependenceLowGrayLevelEmphasis
T2__original_glcm_MCC
T2__original_ngtdm_Contrast
DWI__original_shape_Maximum2DDiameterRow
DWI__original_gldm_GrayLevelNonUniformity
DWI__original_firstorder_Skewness
T1__original_glcm_Imc2
ADC__original_gldm_GrayLevelNonUniformity
e
VOI_P4
4mm_ADC__original_ngtdm_Strength
4mm_T2__original_glcm_Correlation
4mm_DWI__original_firstorder_Skewness
4mm_FLAIR__original_glcm_Idmn
4mm_T1__original_gldm_GrayLevelNonUniformity
4mm_T1__original_gldm_SmallDependenceLowGrayLevelEmphasis
f
VOI_P5
5mm_DWI__original_firstorder_Skewness
5mm_FLAIR__original_glcm_Idmn
5mm_ADC__original_ngtdm_Strength
5mm_ADC__original_gldm_GrayLevelNonUniformity
5mm_T1__original_glcm_Imc2

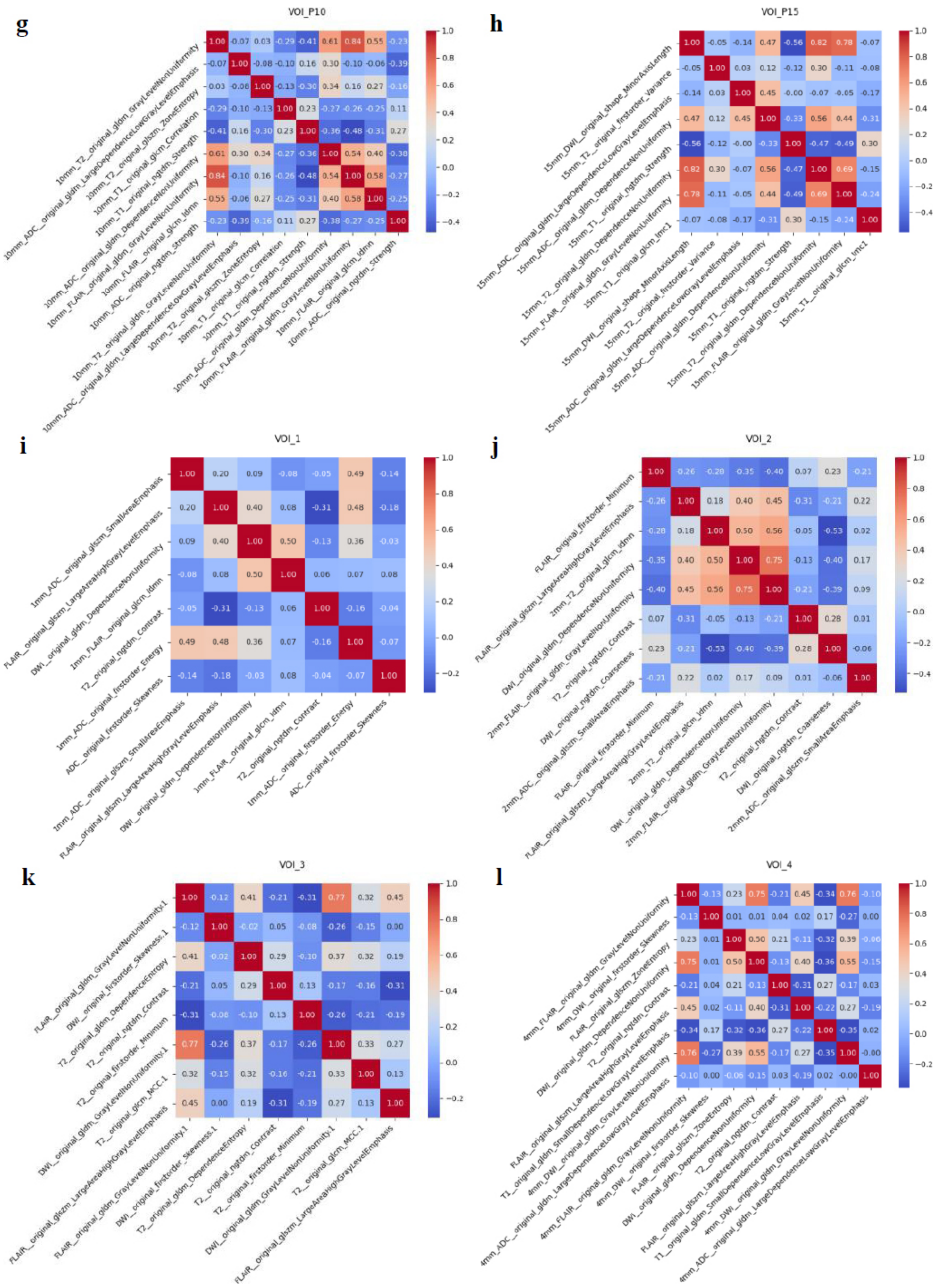
g
VOI_P10
h
VOI_P15
i
VOI_1
j
VOI_2
k
VOI_3
l
VOI_4

m

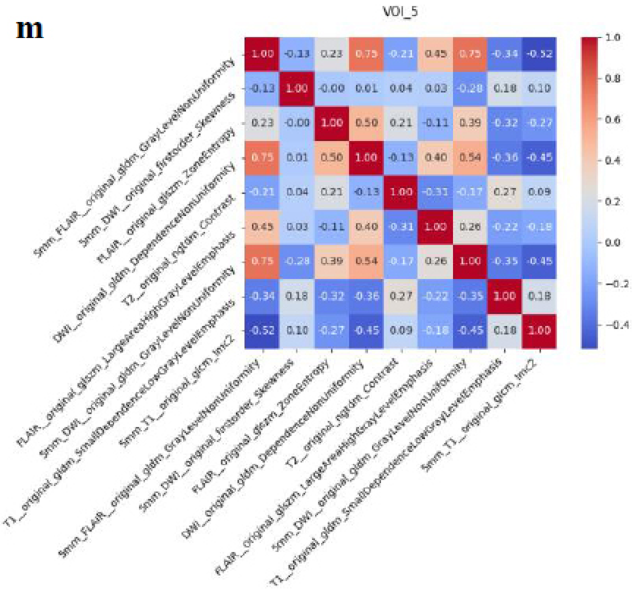

n

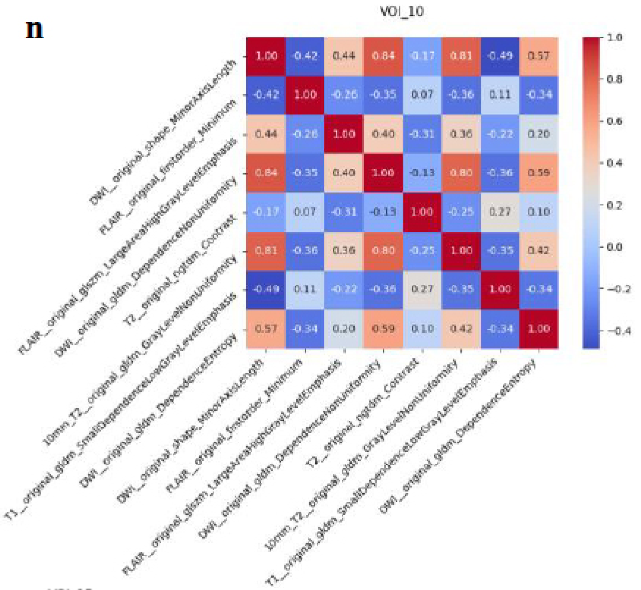

o

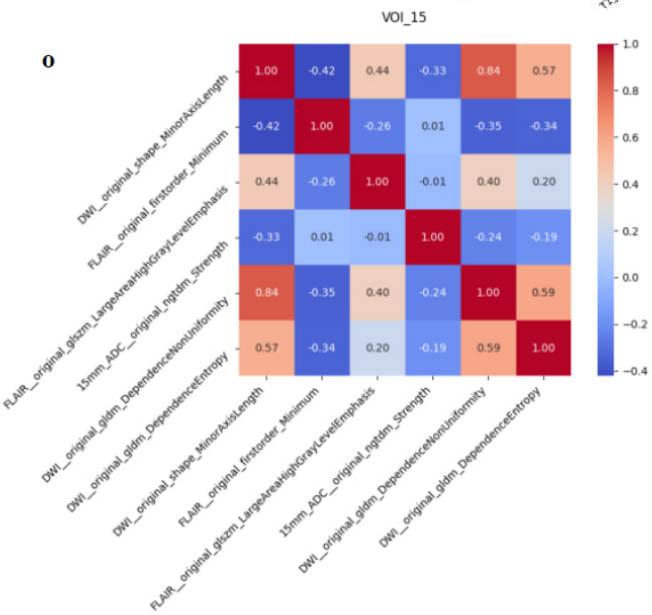

Fig. (S2). Use the LASSO method to retain all features with non-zero coefficients. (**a**): the intratumoral volumes (VOI_I); (**b-h**): peritumoral volumes (VOI_P1-5, 10, 15); (**i-o**): combined category integrating both intratumoral and peritumoral regions(VOI_1-5, 10, 15).

a

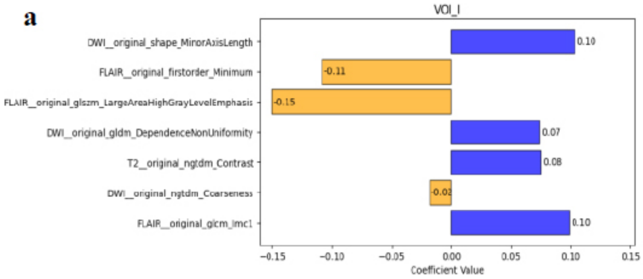

b

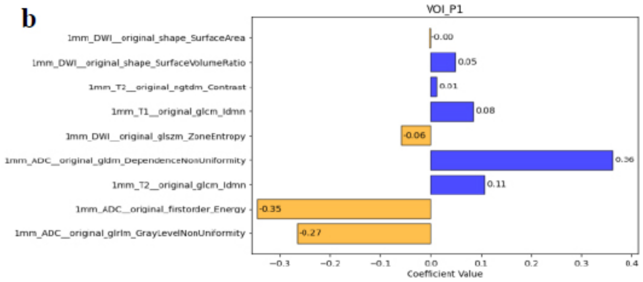

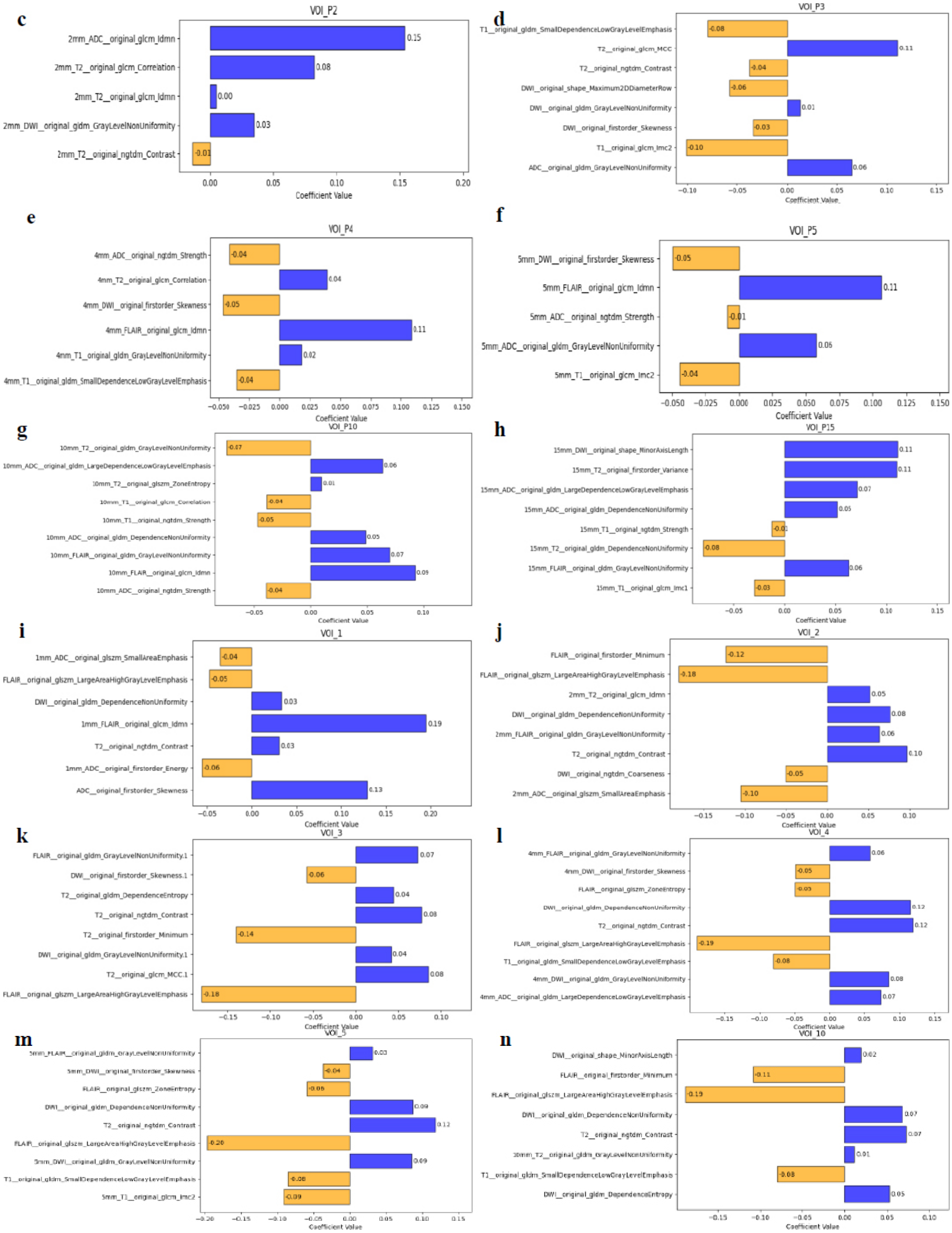
c
VOI_P2
2mm_ADC__original_glcm_Idmn
2mm_T2__original_glcm_Correlation
2mm_T2__original_glcm_Idmn
2mm_DWI__original_gldm_GrayLevelNonUniformity
2mm_T2__original_ngtdm_Contrast
0.15
0.08
0.00
0.03
-0.01
Coefficient Value
d
VOI_P3
T1__original_gldm_SmallDependenceLowGrayLevelEmphasis
T2__original_glcm_MCC
T2__original_ngtdm_Contrast
DWI__original_shape_Maximum2DDiameterRow
DWI__original_gldm_GrayLevelNonUniformity
DWI__original_firstorder_Skewness
T1__original_glcm_Imc2
ADC__original_gldm_GrayLevelNonUniformity
-0.08
0.11
-0.04
-0.06
0.01
-0.03
-0.10
0.06
Coefficient Value
e
VOI_P4
4mm_ADC__original_ngtdm_Strength
4mm_T2__original_glcm_Correlation
4mm_DWI__original_firstorder_Skewness
4mm_FLAIR__original_glcm_Idmn
4mm_T1__original_gldm_GrayLevelNonUniformity
4mm_T1__original_gldm_SmallDependenceLowGrayLevelEmphasis
-0.04
0.04
-0.05
0.11
0.02
-0.04
Coefficient Value
f
VOI_P5
5mm_DWI__original_firstorder_Skewness
5mm_FLAIR__original_glcm_Idmn
5mm_ADC__original_ngtdm_Strength
5mm_ADC__original_gldm_GrayLevelNonUniformity
5mm_T1__original_glcm_Imc2
-0.05
0.11
-0.01
0.06
-0.04
Coefficient Value
g
VOI_P10
10mm_T2__original_gldm_GrayLevelNonUniformity
10mm_ADC__original_gldm_LargeDependenceLowGrayLevelEmphasis
10mm_T2__original_glszm_ZoneEntropy
10mm_T1__original_glcm_Correlation
10mm_T1__original_ngtdm_Strength
10mm_ADC__original_gldm_DependenceNonUniformity
10mm_FLAIR__original_gldm_GrayLevelNonUniformity
10mm_FLAIR__original_glcm_Idmn
10mm_ADC__original_ngtdm_Strength
-0.07
0.06
0.01
-0.04
-0.05
0.05
0.07
0.09
-0.04
Coefficient Value
h
VOI_P15
15mm_DWI__original_shape_MinorAxisLength
15mm_T2__original_firstorder_Variance
15mm_ADC__original_gldm_LargeDependenceLowGrayLevelEmphasis
15mm_ADC__original_gldm_DependenceNonUniformity
15mm_T1__original_ngtdm_Strength
15mm_T2__original_gldm_DependenceNonUniformity
15mm_FLAIR__original_gldm_GrayLevelNonUniformity
15mm_T1__original_glcm_Imc1
0.11
0.11
0.07
0.05
-0.01
-0.08
0.06
-0.03
Coefficient Value
i
VOI_1
1mm_ADC__original_glszm_SmallAreaEmphasis
FLAIR__original_glszm_LargeAreaHighGrayLevelEmphasis
DWI__original_gldm_DependenceNonUniformity
1mm_FLAIR__original_glcm_Idmn
T2__original_ngtdm_Contrast
1mm_ADC__original_firstorder_Energy
ADC__original_firstorder_Skewness
-0.04
-0.05
0.03
0.19
0.03
-0.06
0.13
Coefficient Value
j
VOI_2
FLAIR__original_firstorder_Minimum
FLAIR__original_glszm_LargeAreaHighGrayLevelEmphasis
2mm_T2__original_glcm_Idmn
DWI__original_gldm_DependenceNonUniformity
2mm_FLAIR__original_gldm_GrayLevelNonUniformity
T2__original_ngtdm_Contrast
DWI__original_ngtdm_Coarseness
2mm_ADC__original_glszm_SmallAreaEmphasis
-0.12
-0.18
0.05
0.08
0.06
0.10
-0.05
-0.10
Coefficient Value
k
VOI_3
FLAIR__original_gldm_GrayLevelNonUniformity.1
DWI__original_firstorder_Skewness.1
T2__original_gldm_DependenceEntropy
T2__original_ngtdm_Contrast
T2__original_firstorder_Minimum
DWI__original_gldm_GrayLevelNonUniformity.1
T2__original_glcm_MCC.1
FLAIR__original_glszm_LargeAreaHighGrayLevelEmphasis
0.07
-0.06
0.04
0.08
-0.14
0.04
0.08
-0.18
Coefficient Value
l
VOI_4
4mm_FLAIR__original_gldm_GrayLevelNonUniformity
4mm_DWI__original_firstorder_Skewness
FLAIR__original_glszm_ZoneEntropy
DWI__original_gldm_DependenceNonUniformity
T2__original_ngtdm_Contrast
FLAIR__original_glszm_LargeAreaHighGrayLevelEmphasis
T1__original_gldm_SmallDependenceLowGrayLevelEmphasis
4mm_DWI__original_gldm_GrayLevelNonUniformity
4mm_ADC__original_gldm_LargeDependenceLowGrayLevelEmphasis
0.06
-0.05
-0.05
0.12
0.12
-0.19
-0.08
0.08
0.07
Coefficient Value
m
VOI_5
5mm_FLAIR__original_gldm_GrayLevelNonUniformity
5mm_DWI__original_firstorder_Skewness
FLAIR__original_glszm_ZoneEntropy
DWI__original_gldm_DependenceNonUniformity
T2__original_ngtdm_Contrast
FLAIR__original_glszm_LargeAreaHighGrayLevelEmphasis
5mm_DWI__original_gldm_GrayLevelNonUniformity
T1__original_gldm_SmallDependenceLowGrayLevelEmphasis
5mm_T1__original_glcm_Imc2
0.03
-0.04
-0.06
0.09
0.12
-0.20
0.09
-0.08
-0.09
Coefficient Value
n
VOI_10
DWI__original_shape_MinorAxisLength
FLAIR__original_firstorder_Minimum
FLAIR__original_glszm_LargeAreaHighGrayLevelEmphasis
DWI__original_gldm_DependenceNonUniformity
T2__original_ngtdm_Contrast
10mm_T2__original_gldm_GrayLevelNonUniformity
T1__original_gldm_SmallDependenceLowGrayLevelEmphasis
DWI__original_gldm_DependenceEntropy
0.02
-0.11
-0.19
0.07
0.07
0.01
-0.08
0.05
Coefficient Value

o

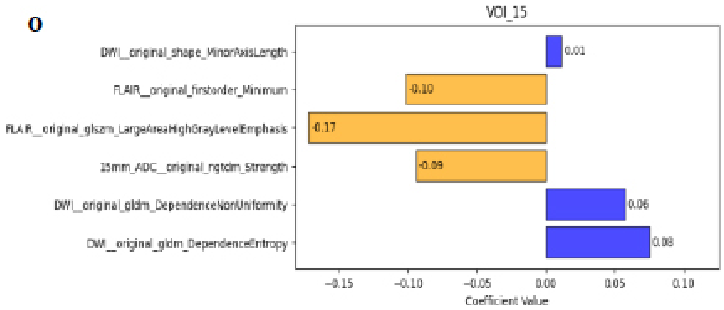

Fig. (S3). The final selected radiomic features and their importance in each VOI. (**a**): the intratumoral volumes (VOI_I); (**b**-**h**): peritumoral volumes (VOI_P1-5, 10, 15); (**i**-**o**): combined category integrating both intratumoral and peritumoral regions(VOI_1-5, 10, 15).

© 2025 The Author(s). Published by Bentham Science Publisher.

This is an open access article distributed under the terms of the Creative Commons Attribution 4.0 International Public License (CC-BY 4.0), a copy of which is available at: https://creativecommons.org/licenses/by/4.0/legalcode. This license permits unrestricted use, distribution, and reproduction in any medium, provided the original author and source are credited.
